# Efficacy and safety of eptinezumab in adults with chronic migraine and medication-overuse headache who also received patient education: 24-week results of the randomized RESOLUTION trial

**DOI:** 10.1186/s10194-026-02423-x

**Published:** 2026-06-13

**Authors:** Henrik W. Schytz, Rigmor H. Jensen, Christofer Lundqvist, Cristina Tassorelli, Fabrizio Vernieri, Michel Lantéri-Minet, Gisela M. Terwindt, Andrew Blumenfeld, Stewart J. Tepper, Mette Krog Josiassen, Gary Jansson, Anders Ettrup, Aurélia Mittoux, Richard B. Lipton

**Affiliations:** 1 Danish Headache Center, Department of Neurology, Rigshospitalet-Glostrup, University of Copenhagen, Copenhagen, Denmark; 2 Departments of Neurology and Health Services Research, Akershus University Hospital, Lørenskog, Norway; 3 Institute of Clinical Medicine, University of Oslo, Oslo, Norway; 4 Department of Brain and Behavioral Sciences, University of Pavia, Pavia, Italy; 5 IRCCS C. Mondino Foundation, Pavia, Italy; 6 Unit of Headache and Neurosonology, Fondazione Policlinico Campus Bio-Medico, Rome, Italy; 7 Department of Medicine and Surgery, Università Campus Bio-Medico di Roma, Rome, Italy; 8 Pain Department, UR2CA, FHU InovPain, Côte Azur University and Centre Hospitalier Universitaire de Nice, Nice, France; 9 INSERM U1107 Migraine and Trigeminal Pain, Auvergne University, Clermont-Ferrand, France; 10 Department of Neurology, Leiden University Medical Centre, Leiden, Netherlands; 11 The Los Angeles Headache Center, Los Angeles, CA USA; 12 The New England Institute for Neurology and Headache, Stamford, CT USA; 13 H. Lundbeck A/S, Copenhagen, Denmark; 14 Department of Neurology and Montefiore Headache Center, Albert Einstein College of Medicine, New York, NY USA

**Keywords:** Eptinezumab, Patient education, Patient-reported outcomes, Chronic migraine, Medication overuse headache, Sustained response

## Abstract

**Background:**

The phase 4 RESOLUTION trial showed that the first 12 weeks of treatment with eptinezumab, an anti-calcitonin gene-related peptide monoclonal antibody, reduced migraine frequency, severity, and disease burden, and improved quality of life (QOL) versus placebo in participants with chronic migraine (CM) and medication-overuse headache (MOH) who also received patient education. Here, we present 24-week eptinezumab efficacy and safety in the RESOLUTION trial.

**Methods:**

RESOLUTION was a randomized, parallel-group, multinational clinical trial that included a 12-week double-blind, placebo-controlled period and a 12-week open-label extension period (OLE). Adults (18–75 years) with CM and MOH (excluding opioid-overuse headache) received a brief educational intervention about MOH at baseline and were randomized (1:1) to IV eptinezumab 100 mg or placebo. At Week 12, all participants received eptinezumab 100 mg. Measures used for primary and key secondary efficacy endpoints were also captured during the OLE: mean changes from baseline in monthly migraine days (primary endpoint: Weeks 1–4), monthly headache days, monthly days with acute medication use, average daily pain, and participants no longer meeting threshold criteria for CM nor MOH. Secondary endpoints (including patient-reported-outcomes [PROs] assessing disease-related burden and health-related QOL) and treatment-emergent adverse events (TEAEs) were also captured during the OLE.

**Results:**

Of 608 participants randomized, 593 (97.5%) were treated with eptinezumab in the OLE, and 584/608 (96.1%) completed the trial. Reductions in migraine frequency and active CM/MOH diagnosis, and improvements across multiple PROs observed in *post hoc* analyses during the placebo-controlled period were sustained during the OLE for participants initially treated with eptinezumab, with similar levels of improvement gained for those initially receiving placebo. The proportion of participants with TEAEs in the OLE was similar between eptinezumab–eptinezumab and placebo–eptinezumab treatment sequence groups (30% vs 34%); no new safety signals were identified.

**Conclusions:**

In participants with CM and MOH who received patient education, reductions in disease burden and improvements in QOL during the first 12 weeks with eptinezumab treatment were sustained for up to 24 weeks following a second eptinezumab infusion, with similar improvements observed in participants switched from placebo to eptinezumab. Eptinezumab was generally well tolerated, with no new safety signals.

**Trial registration:**

ClinicalTrials.gov Identifier: NCT05452239 (https://clinicaltrials.gov/study/NCT05452239); EudraCT Number: 2021-003049-40 (https://www.clinicaltrialsregister.eu/ctr-search/search?query=2021-003049-40)

**Supplementary Information:**

The online version contains supplementary material available at 10.1186/s10194-026-02423-x.

## Graphical Abstract

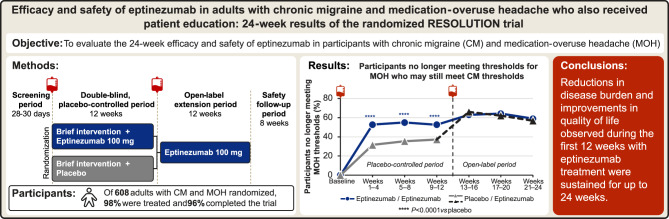

## Introduction

Migraine is a common and disabling neurological disorder [1]; an increased frequency of monthly headache days (MHDs) is associated with a reduced quality of life [1–4]. Acute medications are commonly prescribed for migraine, but they may not sufficiently control migraine, and may decrease in effectiveness over time with frequent use [5]. Progressively increasing use of acute medications is one of the risk factors associated with the progression of episodic migraine (EM) to chronic migraine (CM) and with the development of medication-overuse headache (MOH) [1, 6, 7], a prevalent disorder occurring in approximately 60 million people worldwide [8, 9]. Given the high prevalence of CM and MOH [10, 11], long-term migraine preventive treatment strategies should both reduce burden and prevent progression [7].

According to the International Classification of Headache Disorders, 3rd edition (ICHD-3), MOH is diagnosed when a pre-existing headache disorder develops a new or worsened headache occurring on ≥15 days/month with regular overuse of acute headache drugs for over 3 months [2, 12]. MOH usually develops in people with migraine as their primary headache and is commonly diagnosed among those diagnosed with CM [8, 10]. Negative outcomes for an individual with MOH include decreased health-related quality of life, increased life impact due to headaches, reduced productivity, and frequent healthcare use [10, 11]. The International Headache Society thus recommends the early initiation of migraine preventive treatment to meet the long-term goals of preventing migraine progression, including the progression to MOH, rather than only preventing migraine attacks [7].

Clinical strategies for the treatment of MOH include patient education, medication withdrawal alone, preventive treatment alone, and the combination of medication withdrawal with preventive therapy [8, 10, 13]. European Academy of Neurology guidelines currently recommend education about MOH as the primary treatment approach, followed by medication withdrawal, with preventive treatment added if education and withdrawal are not sufficiently effective [14]. In North America and in European countries with clinical practices that differ from the guidelines of the European Academy of Neurology, it is recommended that education and preventive medication both be provided at the time of MOH presentation [7, 15, 16].

Eptinezumab is a high-affinity, intravenously administered anti–calcitonin gene-related peptide (CGRP) monoclonal antibody (mAb) approved for migraine prevention, offering rapid CGRP inhibition and full bioavailability by the end of infusion [17–19]. In *post hoc* analyses of the PROMISE-2 trial, eptinezumab demonstrated reduced migraine frequency, acute medication use frequency, and disease-related burden in individuals with CM and MOH [20–22].

The RESOLUTION trial (NCT05452239) was the first randomized controlled trial to investigate the combination of anti-CGRP mAb treatment with brief patient education, and the first anti-CGRP preventive trial to report a primary endpoint as early as Week 4 [23–25]. The trial demonstrated the efficacy and safety of eptinezumab versus placebo over 4 and 12 weeks of treatment in participants with CM and MOH who also received a brief educational intervention (BEI) about MOH and how to stop or reduce the overuse of acute medications [25]. The primary results over the first 12 weeks of treatment suggest that initiating eptinezumab treatment in combination with patient education is an effective approach that does not first require medication withdrawal for treating CM and MOH [25, 26]. This paper focuses on the 24-week data, which include the evaluation of (1) the efficacy and safety of eptinezumab in combination with BEI compared to placebo with BEI for the prevention of migraine and treatment of MOH during the 12-week placebo-controlled period, and (2) the effectiveness and safety of eptinezumab during 12-week open-label extension period (OLE).

## Methods

RESOLUTION was a multi-center parallel-group, double-blind, randomized, placebo-controlled phase 4 clinical trial conducted at 76 sites across 11 countries (Australia, Denmark, France, Georgia, Germany, Italy, the Netherlands, Norway, Spain, Sweden, and the United States) from 1 July 2022 to 13 March 2025. This report describes the combined 24-week results from the 12-week double-blind, placebo-controlled period and 12-week OLE, and follows the Consolidated Standards of Reporting Trials (CONSORT) guidelines for randomized controlled trials. The detailed methodology has been published, including the protocol and statistical analysis plan [25, 27]. The trial was conducted in accordance with the Declaration of Helsinki, Good Clinical Practice, and applicable regulatory requirements. The appropriate ethics committee or institutional review board for each site approved the trial; all participants provided written informed consent before trial participation. The RESOLUTION trial is registered with ClinicalTrials.gov (NCT05452239) and EudraCT (2021-003049-40). EudraCT Number obtained: 25 May 2021. ClinicalTrials.gov Identifier obtained: 6 July 2022. First patient enrolled: 1 July 2022.

### Trial design and interventions

In addition to a 4-week screening period, the trial included a placebo-controlled treatment period (12 weeks), an OLE period (12 weeks), and an 8-week safety follow-up (Supplemental Fig. 1). After the screening period, eligible participants received an ~10-minute BEI about MOH and how to stop or reduce the overuse of acute medications [25] and were randomized in a 1:1 ratio to receive a 30–45-minute intravenous (IV) infusion of eptinezumab 100 mg or placebo at the baseline visit (Day 0); the BEI was performed by trained clinicians at the baseline visit only and prior to infusion. At the end of Week 12, all participants received eptinezumab 100 mg during the OLE.

The two treatment sequence groups for the OLE included participants who continued eptinezumab (eptinezumab–eptinezumab; i.e., participants who received eptinezumab at Baseline [Day 0] and end of Week 12) and those who switched from placebo to eptinezumab (placebo–eptinezumab; i.e., participants who received placebo at baseline and eptinezumab at end of Week 12). Participants were randomized with stratification by country and number of previous preventive treatment failures (≤2 vs >2) over the past 5 years before baseline.

Screening visit, IV infusion visits (baseline visit and Week 12 visit), and end-of-trial Week 24 visit occurred on-site; all other visits occurred monthly through telephone or telemedicine. Participants completed a daily electronic diary (eDiary) reporting migraine characteristics and acute headache medication intakes (headache eDiary) and at predefined time points for electronic patient-reported outcomes throughout the trial period to assess endpoints; completion of the eDiary was monitored regularly to ensure adherence between contacts.

### Eligibility criteria

Full exclusion and inclusion criteria, as well as disallowed or restricted concomitant medications, are detailed in the published protocol [25, 27]. Eligible participants included adults aged 18–75 years (inclusive) with diagnoses of both CM and MOH, as defined by ICHD-3 criteria [2]. Participants were required to have an onset of migraine at or before 50 years of age and ≥12 months before the screening visit, as well as ≥8 monthly migraine days (MMDs) and ≥15 MHDs within three months prior to the screening visit. Eligibility also included regular overuse (>3 months) of ≥1 drug for symptomatic and/or acute treatment of headache, as well as  ≥1 preventive treatment failure within ≤5 years prior to the screening visit. Barbiturates and/or opioid analgesics were only allowed provided that the intakes were ≤4 per month for ≥12 weeks before the screening visit and during the entire trial duration (i.e., opioid and/or barbiturate overuse cases were excluded). Before randomization, participants were required to demonstrate compliance with the eDiary for ≥24 of 28 days.

Key exclusion criteria included previous CGRP-targeting treatment failure (including failure with gepants for acute or preventive treatment); history or diagnosis of other headache disorders; severe psychiatric conditions with symptoms not controlled or not adequately treated for ≥6 months before the screening visit; confounding and clinically significant pain syndromes (i.e., chronic low back pain, fibromyalgia, and complex regional pain syndrome); clinically significant cardiovascular disease; and acute or active temporomandibular disorders.

### Assessments

#### Headache eDiary and electronic patient-reported outcomes

For the assessment of headache and migraine variables, participants completed a daily headache eDiary from the screening visit until end of trial or withdrawal visit. Headache characteristics (including presence of associated symptoms) and intake of acute headache medication (including triptans, ergots, non-opioid analgesics, NSAIDs, non-opioid combination analgesics, and opioids/barbiturates) were assessed with yes/no response options, with the exception of pain intensity, which was rated as none, mild, moderate, or severe. Participants also completed an evening eDiary (a part of the eDiary) on a daily basis to report preventive use of acute headache medications, regardless of whether the participant experienced a headache. The electronic patient-reported outcomes scheduled for the baseline and Week 12 visits were to be completed on-site on the visit date and before IV infusion with eptinezumab or placebo, and electronic patient-reported outcomes scheduled for Week 4 were to be completed on the day of or within three days before the scheduled date. The electronic patient-reported outcomes scheduled for Week 24 were to be completed either on-site or in a remote setting within three days before the scheduled date.

At the screening visit, the participant was trained by trial site staff in eDiary and electronic patient-reported outcome use and compliance. To verify eligibility criteria, baseline values, and eDiary compliance during the screening period, the participant was required to record headache and migraine information (for headache/migraine that was not yet recorded or was ongoing) in the eDiary daily prior to intravenous infusion. The trial site staff evaluated ongoing compliance based on eDiary reporting, with the support of automatic alerts.

#### Electronic patient-reported outcome measures

The electronic patient-reported outcome measures captured in RESOLUTION and reported in this paper include the Patient Global Impression of Change (PGIC) scale and patient-identified most bothersome symptom (PI-MBS) scale to evaluate effect on disease symptomatology and overall disease status; six-item Headache Impact Test (HIT-6), modified Migraine Disability Assessment (mMIDAS), and Migraine-specific Work Productivity and Activity Impairment questionnaire (WPAI:M) to evaluate effect on measures of impact and disability; as well as the Migraine-Specific Quality-of-Life questionnaire v2.1 (MSQ v2.1) and EuroQol EQ-5D visual analogue scale (EQ-5D VAS) to evaluate effect on quality of life.

Participants reported their impression of change in their disease status related to activity limitations, emotions, symptoms, and overall quality of life since the baseline visit after treatment using the PGIC [28]. This single patient-reported item is rated on a seven-point scale (1 = very much improved; 2 = much improved; 3 = minimally improved; 4 = no change; 5 = minimally worse; 6 = much worse; 7 = very much worse). Participants reported the most bothersome symptom including nausea, vomiting, light or sound sensitivity, mental cloudiness, fatigue, pain during activity, mood changes, and other symptoms associated with migraine during the screening visit. Participants rated the symptom improvement after treatment using the PI-MBS, which uses the same seven-point scale as the PGIC [29]. For both measures, a responder was defined as a participant who reported “much improved” or “very much improved” (analyzed *post hoc*).

The HIT-6 questionnaire assesses the impact of headaches on the ability to function in daily life. The HIT-6 contains six questions (three with a four-week recall, three with no specified recall period), with each item rated as “never” to “always”: never (6), rarely (8), sometimes (10), very often (11), and always (13). The HIT-6 total score ranges from 36 to 78 and is the sum of each response score, which is used to grade headache-related impact on life: severe (≥60), substantial (56–59), some (50–55), and little to none (≤49) [30]. A ≥5-point reduction from baseline in HIT-6 total score is considered a clinically meaningful improvement [31, 32].

The mMIDAS questionnaire assesses data of missed activities and days of reduced ability to engage in activities (reduced participation) in multiple domains, including school, work, social, family, and leisure activities over the past four weeks; the total score includes five questions related to these domains [33]. The mMIDAS scale has a one-month recall period, whereas the original MIDAS scale has a three-month recall period [34]. Two additional questions are included in the MIDAS: one on monthly headache frequency and another on pain intensity [34]; however, these questions are not included in the total score. A ≥30% improvement from baseline in mMIDAS total score is considered clinically meaningful when the baseline score is >20 [31].

The WPAI:M consists of six questions that assess activities over the preceding seven days: employment status (one item); number of hours worked, the number of hours missed from work due to the patient’s condition, or due to other reasons (three items); and how much the participant’s condition affects their ability to complete normal daily activities and their productivity at work (two visual numerical scales) [35]. To quantify impairment due to migraine, these questions are used to calculate four domains: absenteeism (percentage of work time missed), presenteeism (percentage of impairment while working), work productivity loss (percentage of overall work impairment), and activity impairment (percentage of activity impairment).

The MSQ v2.1 assesses migraine-related quality of life over the past four weeks. It consists of 14 items that lead to scores in 3 domains, with higher scores indicating better quality of life: role function–restrictive (seven items); role function–preventive (four items); and emotional function (three items). Each item is scored on a six-point scale ranging from 1 (never) to 6 (always); raw domain scores are summed and transformed to a scale ranging from 0 to 100 [36].

Participants used the short visual analogue scale (VAS) assessment from the EQ-5D-5 L instrument to report their current overall health state on the day they completed it. The VAS score ranges from 0 (worst imaginable) to 100 (best imaginable health state) [37].

### Endpoints

The full list of primary, key secondary, other secondary, exploratory, and safety endpoints are summarized in the published protocol [25, 27]. The primary endpoint was change from baseline in number of monthly migraine days (MMDs) during Weeks 1–4 of the 12-week placebo-controlled period (previously reported [25]). Measures for key secondary endpoints that were also captured during the 12-week OLE included the change from baseline in MMDs (Weeks 1–12 and 13–24); change from baseline in MHDs (Weeks 1–4, 1–12, and 13–24); participants no longer meeting threshold criteria for either CM or MOH (Weeks 1–4, 1–12, and 13–24); change from baseline in average daily pain (Weeks 1–2 and 13–24 [*post hoc*: Weeks 1–12]); and change from baseline in monthly days with acute medication use (Weeks 1–4, 1–12, and 13–24). Thresholds for no longer meeting CM/MOH criteria were based on ICHD-3 diagnostic criteria that were confirmed at baseline and are detailed previously in the primary report of RESOLUTION [25].

Measures for secondary endpoints that were also captured during the OLE included: not fulfilling threshold criteria for CM (Weeks 1–4 and 1–12 [*post hoc*: Weeks 13–24]); not fulfilling threshold criteria for MOH (Weeks 1–4 and 1–12 [*post hoc*: Weeks 13–24]); rate of ≥50% reduction from baseline in MMDs (Weeks 1–4 and 1–12 [*post hoc*: Weeks 13–24]); rate of ≥75% reduction from baseline in MMDs (Weeks 1–4 and 1–12 [*post hoc*: Weeks 13–24]); PGIC score at Weeks 4, 12, and 24; and PI-MBS score at Weeks 12 and 24.

Other secondary endpoints included the change from baseline to Weeks 4, 12, and 24 for the following electronic patient-reported outcome measures: HIT-6, mMIDAS, WPAI:M (Weeks 12 and 24 only), MSQ v2.1, and EQ-5D VAS.

All continuous measures were captured across all 4-week and 12-week intervals during the placebo-controlled period and OLE, except for electronic patient-reported outcome measures, which were captured at the specified time points. The safety and tolerability of eptinezumab during the OLE were assessed similarly to the primary report of the placebo-controlled period and included adverse event (AE) monitoring, as well as absolute values, changes from baseline, and potentially clinically significant values for vital signs. Treatment-emergent AEs (TEAEs) during the OLE (defined as any TEAE that began, increased in intensity, or became serious during or following infusion at the end of Week 12) are presented in this paper.

### Statistical analysis

Efficacy and safety endpoints were analyzed using data from the all-participants-treated-open-label set (all randomized participants who received an infusion of eptinezumab in the OLE). For the efficacy analysis, this is to allow a complete trial analysis by combining the placebo-controlled period and OLE. The efficacy analysis of the placebo-controlled period endpoints in these analyses based on the all-participants-treated-open-label set may differ slightly compared to the results for the original analyses presented in the primary reports [25, 26], which were based on the full analysis set (all treated participants who had a valid baseline assessment and had ≥1 valid four-weekly post-baseline assessment of MMDs during Weeks 1–12).

The mixed model for repeated measures (MMRM) for the change from baseline in MMDs included fixed effects for: baseline score as a continuous covariate; treatment group (eptinezumab, placebo), month (months 1, 2, and 3), country, and previous treatment failures (≤2, >2) as factors; and interaction terms for treatment-by-month, previous treatment failures-by-month, and baseline score-by-month. An unstructured covariance matrix was used to model the between and within-participant covariance. For the analysis of the placebo-controlled and OLE periods combined, the MMRM model used for estimation included months 1, 2, 3, 4, 5 and 6. *Post hoc*, this model was also used to test for treatment differences seen within the placebo-controlled period to substantiate that the effects seen in the analysis of the first 3 months were also clearly seen in the population of participants that continued into the OLE. From the combined periods, estimates across 12-week intervals were derived from the MMRM model using equal weights for the three 4-week intervals in each 12-week period. Other continuous key secondary, secondary, and *post hoc* continuous endpoints were analyzed using a similar model for the combined periods, using the corresponding baseline value as a covariate (except for PGIC and PI-MBS, where no baseline value exists). For average daily pain, in addition to the Weeks 1–2 endpoint, results for the change from baseline to each 4-week interval during Weeks 1–12 are presented *post hoc*.

Logistic regression was used for the binary variables (including responder rates), which were analyzed using baseline MMDs as a covariate, with treatment, country, and previous treatment failures (≤2, >2) as categorical variables. *Post hoc* analyses were conducted to evaluate responder rates for PGIC and PI-MBS (i.e., the percentage of participants “much improved” or “very much improved,” calculated for each measure), as well as HIT-6 total score responder rates (≥5-point reduction from baseline in total score [31, 32]) during the placebo-controlled period and OLE. Resulting *post hoc p-*values from statistical testing for differences between eptinezumab and placebo were presented for the placebo-controlled period endpoints. For the OLE endpoints, results were presented using point estimates and standard errors (i.e., no testing for differences between the treatment sequence groups are presented for the OLE). All statistical analyses were done using SAS (version 9.4 or later).

## Results

### Participants

**Fig. 1:**
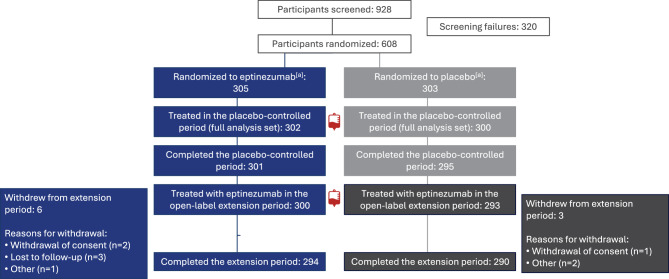
Participant disposition. ^[a]^ All participants received a brief educational intervention at the baseline visit prior to the infusion of eptinezumab or placebo. Efficacy in the placebo-controlled period was analyzed using data from the full analysis set (all participants in the all-participants-treated set who had a valid baseline assessment and ≥1 valid post-baseline 4-week assessment of MMDs across Weeks 1–12). Safety and efficacy in the open-label extension period were analyzed using data from the all-participants-treated-open-label set (all participants in the all-randomized-participants set who received an infusion of eptinezumab in the open-label extension period)

Of the 608 randomized adults who met the eligibility criteria, 596 (98.0%) completed the 12-week placebo-controlled period (eptinezumab 100 mg arm, *n* = 301; placebo arm, *n* = 295) and 584 (96.1%) completed the entire trial. The full analysis data set comprised 602 participants (eptinezumab 100 mg arm, *n* = 302; placebo arm, *n* = 300). The all-participants-treated-open-label set included 593 of 608 (97.5%) randomized participants who received treatment in the 12-week OLE (eptinezumab–eptinezumab treatment group, *n* = 300; placebo–eptinezumab treatment group, *n* = 293). Figure 1 summarizes participant disposition and reasons for withdrawal from the OLE.

Demographics and baseline clinical characteristics in the all-participants-treated-open-label set were similar in the two treatment-sequence groups, with baseline scores indicative of moderate to severe disease-related burden and poor health-related quality of life (Supplemental Table 1).

### Clinical efficacy

#### Migraine and headache frequency, and migraine responder rates

**Fig. 2:**
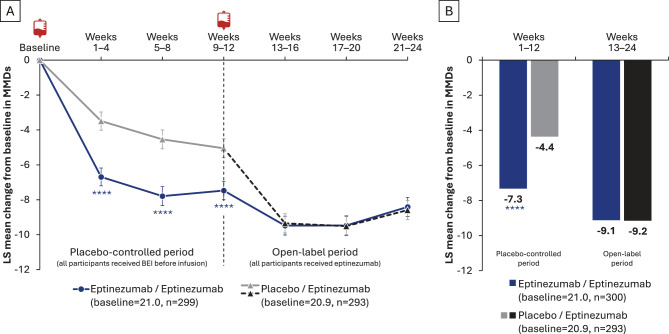
Change from baseline in MMDs over (**A**) 4-week and (**B**) 12-week intervals (all-participants-treated-open-label set). All participants received brief educational intervention before eptinezumab or placebo infusion at baseline; at the end of Week 12, all participants received eptinezumab. Error bars show standard error, and a mixed model for repeated measures was used. *post hoc p-*values for differences between eptinezumab and placebo are presented for the placebo-controlled period. ****p<0.0001 vs placebo. BEI, brief educational intervention; LS, least-squares; MMDs, monthly migraine days

As previously reported, the trial met the primary endpoint, with statistically significantly greater reductions in MMDs from baseline over Weeks 1–4 in the eptinezumab group compared to the placebo group [25]. These effects were maintained across all 4-week intervals to Weeks 9–12 (Fig. 2A). Reductions in MMDs during the placebo-controlled period were sustained during the OLE for the eptinezumab–eptinezumab treatment group (Weeks 13–24, −9.1). For the placebo–eptinezumab treatment group, reductions in MMDs were similar relative to baseline (Weeks 13–24, −9.2) (Fig. 2B).

**Fig. 3:**
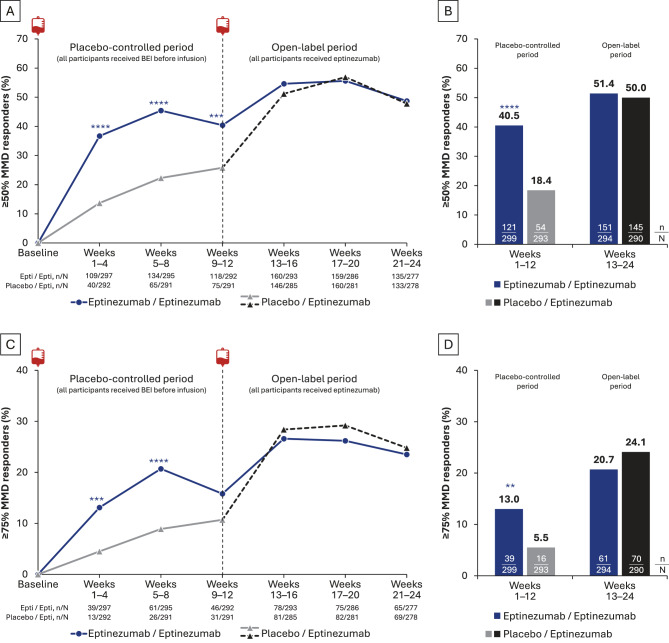
≥50% and ≥75% MMD responder rates over 4-week and 12-week intervals (all-participants-treated-open-label set). The ≥50% MMD responder rates over each (**A**) 4-week and (**B**) 12-week interval, and ≥75% MMD responder rates over each (**C**) 4-week and (**D**) 12-week interval were assessed. Responder rates over Weeks 13–24 were analyzed *post hoc*. All participants received brief educational intervention before eptinezumab or placebo infusion at baseline; at the end of Week 12, all participants received eptinezumab. Participants were ≥50% MMD responders for each 12-week or 4-week interval if they had a ≥50% reduction from baseline in MMDs for the respective interval, and participants were ≥75% MMD responders for each 12-week or 4-week interval if they had a ≥75% reduction from baseline in MMDs for the respective interval. *post hoc p-*values for differences between eptinezumab and placebo are presented for the placebo-controlled period; where a *p*-value is not presented for the placebo-controlled period, the difference was not significant. **p<0.01, ***p<0.001, ****p<0.0001; all vs placebo. BEI, brief educational intervention; Epti, eptinezumab; MMD, monthly migraine day

Changes from baseline in MHDs (Supplemental Figure 2) as well as ≥50% and ≥75% MMD responder rates (Fig. 3) paralleled changes from baseline in MMDs. The placebo–eptinezumab treatment group showed lower responder rates for Weeks 1–12 than the eptinezumab–eptinezumab treatment group, with similar responder rates in both treatment groups in *post hoc* analyses of Weeks 13–24 (Fig. 3).

#### Average daily pain

**Fig. 4:**
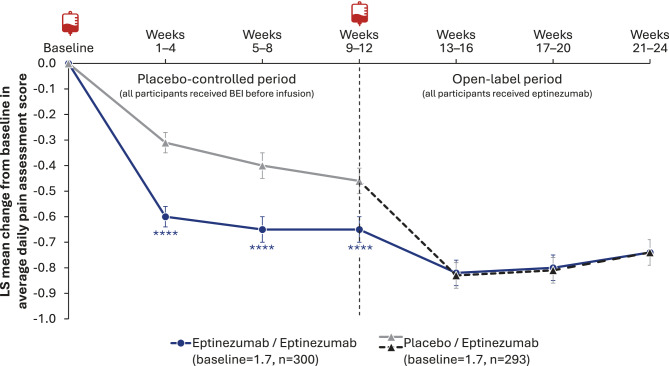
Change from baseline in average daily pain assessment score over 4-week intervals (all-participants-treated-open-label set). All participants received brief educational intervention before eptinezumab or placebo infusion at baseline; at the end of Week 12, all participants received eptinezumab. Pain intensity was rated on a 3-point scale: 1 = mild, 2 = moderate, 3 = severe. A score of 0 was given for days with no headache during the relevant period. Error bars show standard error, and a mixed model for repeated measures was used. Change from baseline in average daily pain across Weeks 1–12 was analyzed *post hoc*. *Post hoc p-*values for differences between eptinezumab and placebo are presented for the placebo-controlled period. ****p<0.0001 vs placebo. BEI, brief educational intervention; LS, least-squares

Change from baseline in average daily pain assessed as none, mild, moderate, or severe on a 4-point scale ranging from 0-3 over Weeks 1–2 was a key secondary endpoint, with results for Weeks 1–2 previously reported [25]. Over Weeks 1–4, a greater reduction in average daily pain was seen in participants initially treated with eptinezumab relative to those initially treated with placebo, with effects generally maintained across all 4-week intervals to Weeks 9–12 as seen in *post hoc* analyses (Fig. 4). Reductions from baseline in average daily pain during the placebo-controlled period were sustained throughout the OLE for the eptinezumab–eptinezumab treatment group, while the placebo–eptinezumab treatment group achieved similar levels of reduction in pain during the OLE.

#### Acute medication use

**Fig. 5:**
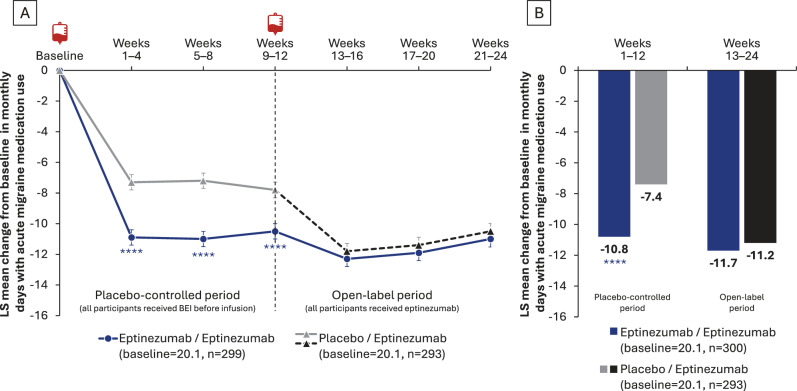
Change from baseline in monthly days with acute medication use (all-participants-treated-open-label set). Change from baseline in monthly days with acute medication use was assessed over (**A**) 4-week and (**B**) 12-week intervals. All participants received brief educational intervention before eptinezumab or placebo infusion at baseline; at the end of Week 12, all participants received eptinezumab. Error bars show standard error, and a mixed model for repeated measures was used. *Post hoc p-*values for differences between eptinezumab and placebo are presented for the placebo-controlled period. ****p<0.0001 vs placebo. BEI, brief educational intervention; LS, least-squares

Eptinezumab was associated with greater reductions than placebo in monthly days with acute medication use across all three 4-week intervals during the placebo-controlled period (Fig. 5A). During the OLE, reductions in acute medication use were sustained for those continuing eptinezumab, while the placebo–eptinezumab treatment group achieved similar changes from baseline as those who started on eptinezumab during the OLE (Fig. 5B).

#### Participants no longer meeting the criteria for CM, for MOH, and for either CM or MOH

**Fig. 6:**
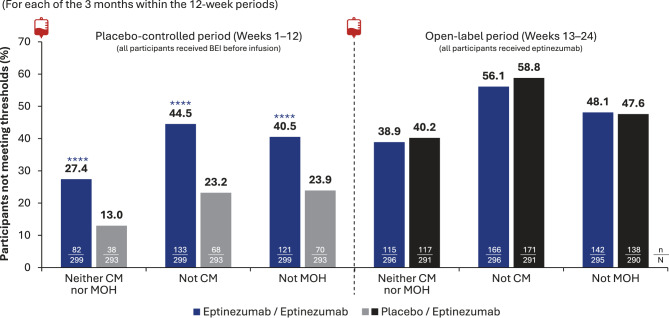
Participants no longer meeting the thresholds for CM/MOH criteria (all-participants-treated-open-label set). The proportions of participants no longer fulfilling the thresholds for CM/MOH criteria were assessed over Weeks 1–12 and Weeks 13–24. All participants received brief educational intervention before eptinezumab or placebo infusion at baseline; at the end of Week 12, all participants received eptinezumab. Thresholds defining CM and MOH were based on ICHD-3 diagnostic criteria for each disease, with CM determined by the monthly frequency of headache days (≥15) and migraine days (≥8) and MOH determined by the monthly frequency of acute medication use (≥10 or ≥15 days depending on medication class). For analyses over 12-week intervals, participants were considered not meeting the thresholds for CM and MOH if they did not meet the thresholds for each of the three 4-week intervals in the 12-week *p*eriod. *Post hoc* endpoints were defined for not fulfilling threshold criteria for CM across Weeks 13–24 and not fulfilling threshold criteria for MOH across Weeks 13–24. *Post hoc p-*values for differences between eptinezumab and placebo are presented for the placebo-controlled period. ****p<0.0001 vs placebo. BEI, brief educational intervention; CM, chronic migraine; ICHD-3, International Classification of headache Disorders, 3rd edition; MOH, medication-overuse headache

Over Weeks 1–4, eptinezumab treatment resulted in a higher proportion of participants no longer fulfilling CM/MOH criteria (i.e., participants no longer meeting the thresholds for CM and MOH, CM, or MOH criteria) compared to placebo. Over 4-week intervals, results for the eptinezumab–eptinezumab treatment group were generally maintained during the placebo-controlled period and were generally sustained during the OLE, and the proportions of participants no longer meeting CM/MOH criteria were similar in both treatment groups during the OLE (Supplemental Figure 3). Over Weeks 1–12, eptinezumab treatment resulted in a higher proportion of participants no longer meeting CM/MOH criteria compared to placebo. Over Weeks 13–24, the proportion of participants no longer meeting CM/MOH criteria were sustained relative to the preceding dosing interval for those continuing eptinezumab, with similar proportions seen in both treatment groups (Fig. 6).

### Patient-reported outcomes

As previously reported [25, 26], eptinezumab treatment also demonstrated a positive impact on electronic patient-reported outcomes during the 12-week placebo-controlled period. Sustained outcomes were seen at Week 24 in the eptinezumab–eptinezumab treatment group, with the placebo–eptinezumab treatment group achieving similar outcomes at Week 24 as those who started on eptinezumab (Supplemental Table 1).

#### Effect on disease symptomatology and overall disease status

**Fig. 7:**
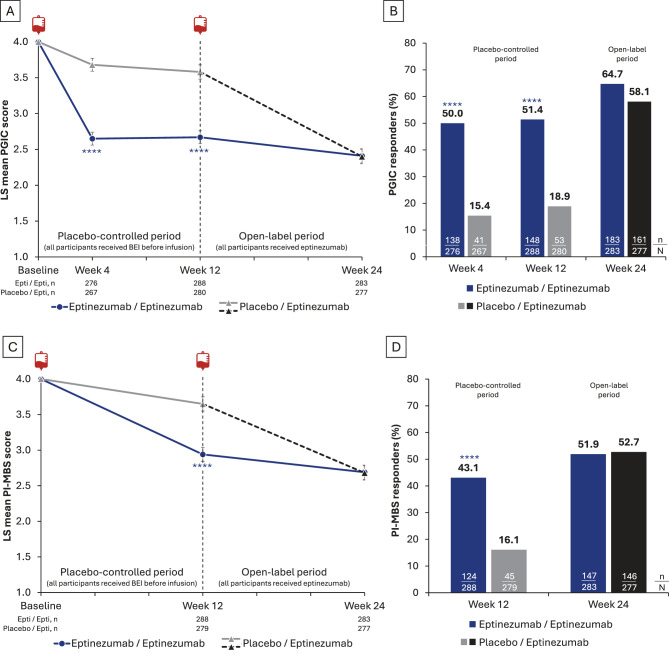
Effect on disease symptomatology and overall disease status (all-participants-treated-open-label set). PGIC: (**A**) Mean score and (**B**) Responder rates, and PI-MBS: (**C**) Mean score and (**D**) Responder rates. All participants received brief educational intervention before eptinezumab or placebo infusion at baseline; at the end of Week 12, all participants received eptinezumab. Both the PGIC and PI-MBS rating scales range from 1 (very much improved) to 7 (very much worse). (**A**) The representative baseline value of 4 (no change) is deterministic and not based on individual data at baseline. Error bars show standard error, and mixed model for repeated measures was used. (**B**) Participants were PGIC responders if they scored 1 (very much improved) or 2 (much improved) on the PGIC scale (analyzed *post hoc*); a logistic regression model was used. (**C**) The representative baseline value of 4 (no change) is deterministic and not based on individual data at baseline. Error bars show standard error, and mixed model for repeated measures was used. (**D**) Participants were PI-MBS responders if they scored 1 (very much improved) or 2 (much improved) on the PI-MBS scale (analyzed *post hoc*); a logistic regression model was used. In all panels, *post hoc p-*values for differences between eptinezumab and placebo are presented for the placebo-controlled period. ****p<0.0001 vs placebo. BEI, brief educational intervention; Epti, eptinezumab; LS, least-squares; PGIC, patient Global impression of Change; PI-MBS, patient-identified most bothersome symptom

Mean PGIC scores were lower with eptinezumab than with placebo at Week 12. At Week 24, PGIC improvements were sustained for those continuing eptinezumab, while the placebo–eptinezumab treatment group achieved similar values as the eptinezumab–eptinezumab treatment group (Fig. 7A). The percent of PGIC responders (i.e., those who scored “very much improved” or “much improved” on the PGIC scale; analyzed *post hoc*) was greater with eptinezumab than with placebo at Week 12. At Week 24, PGIC responder rates were similar in both treatment groups (Fig. 7B).

Results for mean PI-MBS scores (Fig. 7C) and PI-MBS responder rates (analyzed *post hoc*) (Fig. 7D) were similar to those seen for PGIC scores.

#### Effect on measures of impact and disability

**Fig. 8:**
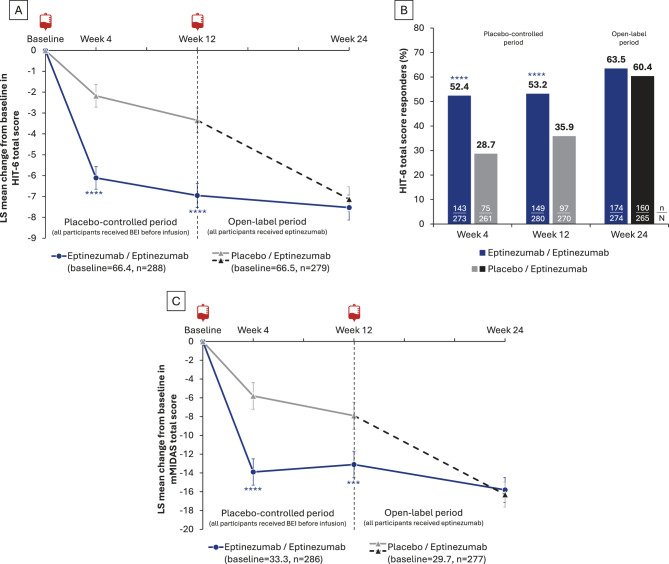
Effect on measures of impact and disability (all-participants-treated-open-label set). (**A**) Change from baseline in HIT-6 total score, (**B**) HIT-6 total score responder rate (defined as proportion of participants with a ≥5-point reduction from baseline in HIT-6 total score), and (**C**) Change from baseline in mMIDAS total score. All participants received brief educational intervention before eptinezumab or placebo infusion at baseline; at the end of Week 12, all participants received eptinezumab. (**A**) and (**C**) Error bars show standard error, and a mixed model for repeated measures was used. (**B**) A logistic regression model was used. (**C**) The mMIDAS has a one-month recall period and is a modified version of the original MIDAS, which has a three-month recall period [34]. A ≥30% improvement from baseline in mMIDAS total score is considered clinically meaningful [31], with the threshold for clinically meaningful reduction from baseline at approximately −10.0 in the eptinezumab arm and −8.9 in the placebo arm. *Post hoc p-*values for differences between eptinezumab and placebo are presented for the placebo-controlled period. ***p<0.001, ****p<0.0001; both vs placebo. BEI, brief educational intervention; HIT-6, 6-item headache impact Test; LS, least-squares; MIDAS, migraine disability Assessment; mMIDAS, modified migraine disability assessment

At Weeks 4 and 12, HIT-6 total scores improved more in the eptinezumab arm than in the placebo arm (Fig. 8A), with a greater proportion of participants in the eptinezumab arm reporting a clinically meaningful change from baseline (i.e., ≥5-point improvement [31, 32]) (Fig. 8B). At Week 24, mean HIT-6 total scores were reduced by a similar extent across both treatment groups, with similar HIT-6 total score responder rates.

At Weeks 4 and 12, the mMIDAS total score improved more in the eptinezumab arm than in the placebo arm, with a clinically meaningful change from baseline in mean score with eptinezumab (i.e., >30% improvement [31], corresponding to approximate mean reductions from baseline of ‑10.0 in the eptinezumab–eptinezumab treatment group and −8.9 in the placebo–eptinezumab treatment group) (Fig. 8C). At Week 24, mMIDAS total scores were reduced by a similar extent across both treatment groups.

At Week 12, the eptinezumab arm had a more favorable improvement than the placebo arm in WPAI:M sub-scores, except for WPAI:M absenteeism. At Week 24, reductions for all WPAI:M sub-scores were sustained in the eptinezumab–eptinezumab treatment group, and the placebo–eptinezumab treatment group demonstrated a similar reduction from baseline as those who were started on eptinezumab (Supplemental Figure 4). From Week 12 to Week 24, the eptinezumab–eptinezumab treatment group appeared to experience greater reductions in WPAI:M absenteeism sub-scores compared to the other three sub-scores.

#### Effect on quality of life

**Fig. 9:**
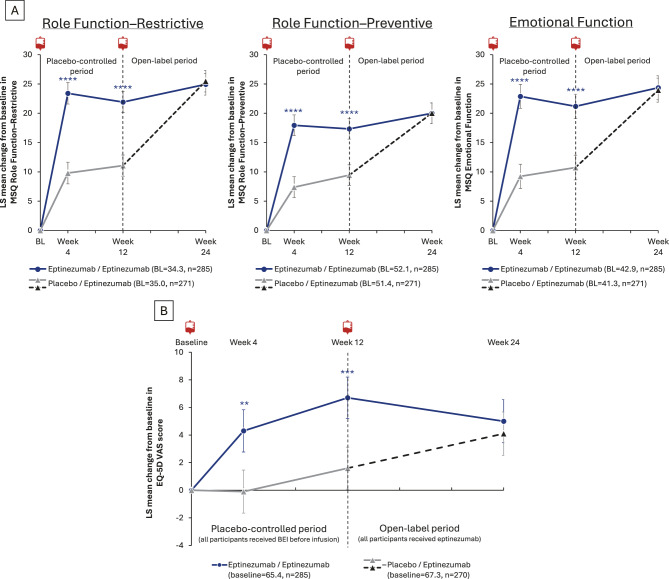
Effect on quality of life (all-participants-treated-open-label set). (**A**) Change from baseline in MSQ v2.1 domain scores, and (**B**) Change from baseline in EQ-5D VAS score. All participants received brief educational intervention before eptinezumab or placebo infusion at baseline; at the end of Week 12, all participants received eptinezumab. Error bars show standard error, and mixed model for repeated measures was used. *Post hoc p-*values for differences between eptinezumab and placebo are presented for the placebo-controlled period. **p<0.01, ***p<0.001, ****p<0.0001; all vs placebo. BEI, brief educational intervention; BL, baseline; LS, least-squares; MSQ v2.1, Migraine-Specific quality of life questionnaire, version 2.1; VAS, visual analog scale

The increases in MSQ v2.1 domain scores (Fig. 9A) and EQ-5D VAS score (Fig. 9B) at Week 12 showed that eptinezumab improved quality of life more than placebo, with similar increases from baseline observed in both treatment groups at Week 24.

### Safety and tolerability

The proportion of participants with TEAEs across the extension period was similar in the two treatment groups (eptinezumab–eptinezumab, 30%; placebo–eptinezumab, 34%) (Table 1). Most TEAEs during the OLE were mild to moderate, and few led to treatment withdrawal (0 participants in the eptinezumab–eptinezumab treatment group and 5/293 [1.7%] participants in the placebo–eptinezumab treatment group) or infusion interruption (1/300 [0.3%] participants in the eptinezumab–eptinezumab treatment group and 1/293 [0.3%] participants in the placebo–eptinezumab treatment group). The most commonly reported TEAEs (≥2% incidence in any treatment sequence group) were nasopharyngitis and influenza-like symptoms. The proportion of participants with serious AEs, severe TEAEs, TEAEs leading to withdrawal from treatment, and TEAEs leading to infusion interruption were low, all <2%, with similar incidences in the treatment sequence groups. AEs of special interest, including hypersensitivity and anaphylactic reactions, cardiovascular/cerebrovascular events, and AEs potentially associated with trial infusion, and vital signs did not show new safety signals.

**Table 1:** Summary of treatment-emergent adverse events in the open-label extension period (all-participants-treated-open-label set)

	Eptinezumab/ **Eptinezumab**^[a]^ (n = 300)	Placebo/ **Eptinezumab**^[a]^ (n = 293)
**Any TEAE**, n (%)	91 (30.3)	101 (34.5)
**Any TEAE by intensity**, n (%)		
Mild	47 (15.7)	56 (19.1)
Moderate	40 (13.3)	41 (14.0)
Severe	4 (1.3)	4 (1.4)
**Any treatment-emergent SAE**, n (%)	2 (0.7)	5 (1.7)
**Any TEAE leading to withdrawal from treatment**, n (%)	0	5 (1.7)
**Any TEAE leading to infusion interruption**, n (%)	1 (0.3)	1 (0.3)
**Most common TEAEs (incidence of ≥2% in either treatment sequence)**, n (%)		
Nasopharyngitis	7 (2.3)	10 (3.4)
Influenza	7 (2.3)	6 (2.0)

All participants received a brief educational intervention before eptinezumab or placebo infusion at baseline; at the end of Week 12, all participants received eptinezumab. Data represent the number (percentage) of participants. SAE, serious adverse event; TEAE, treatment-emergent adverse event

## Discussion

Results from the open-label extension of the phase 4 RESOLUTION trial showed sustained benefits of eptinezumab for up to 24 weeks in participants with CM and MOH who received patient education about MOH at baseline, including reduced migraine frequency, fewer participants meeting criteria for CM and MOH, and improved patient-reported outcomes related to disease burden and quality of life. Across efficacy and patient-reported outcomes, improvements observed during the 12-week placebo-controlled period were generally sustained during the 12-week extension period of open-label eptinezumab treatment for participants initially treated with eptinezumab, with similar levels of improvement confirmed for those who initially had received placebo. The rapid and consistent improvements seen after participants switched from placebo to open-label eptinezumab treatment were consistent with the findings from the placebo-controlled period, showing treatment effects of eptinezumab also among those who originally had received placebo.

In RESOLUTION, eptinezumab with patient education was superior to placebo with patient education in reducing MMDs and MHDs, in increasing the proportion of participants who no longer fulfilled the threshold for CM/MOH criteria, and in reducing average daily pain and acute medication use, with improvements observed as early as Weeks 1–4 and consistent across Weeks 1–12. In the OLE, improvements seen with eptinezumab were sustained across Weeks 13–24 for those who received a second eptinezumab infusion, and the placebo group generally demonstrated rapid and similar levels of improvement after receiving eptinezumab.

Patient-reported outcomes are valuable for evaluating the impact of CM and MOH on associated disability and health-related quality of life [22, 38]. During the placebo-controlled period of RESOLUTION, eptinezumab with patient education demonstrated greater improvements than placebo with patient education in patient-reported overall clinical impression of change, impact and burden of migraine, migraine-related work productivity and activity impairment, and migraine-specific and overall health-related quality of life [26]. Most patient-reported outcomes showed a similar pattern of rapid, clinically meaningful improvements (i.e., ≥5-point improvement in HIT-6 total score; >30% improvement in mMIDAS total score; and MSQ v2.1 sub-score differences in role function – restrictive [>3.2], preventive [>4.6], and emotional function [>7.5] [31, 32, 39]) with eptinezumab during the placebo-controlled period, which were generally sustained during the OLE. The group initially treated with placebo improved similarly to the group initially treated with eptinezumab when they received open-label eptinezumab treatment during the extension period.

The effect of open-label eptinezumab treatment seen in participants initially treated with placebo suggests that while patient education alone may have some effect, together with the well-known placebo response normally observed in randomized, placebo-controlled trials on CM and CM/MOH [38, 40], patient education alone is not sufficient to obtain the rapid improvement observed in the eptinezumab group during the placebo-controlled period.

Eptinezumab was well tolerated, with no new safety signals identified during the OLE compared to the placebo-controlled period or to previous trials [17]. Sustained improvements seen with eptinezumab in RESOLUTION are consistent with the sustained migraine-preventive benefits of eptinezumab seen across the 24-week PROMISE2 trial in adults with CM, and during the 18-month DELIVER trial in participants with migraine for whom 2–4 prior nonspecific preventive treatments had failed [41, 42].

Prior clinical trials have demonstrated efficacy of patient education in reducing medication use for people with MOH [43–45]. As patient education alone is often an inadequate treatment [13], this trial was designed to assess the benefit of eptinezumab when added to patient education in people with CM and MOH. The incremental benefits of eptinezumab with patient education over placebo with patient education, as well as the sustained improvements seen with eptinezumab, suggest that adding eptinezumab to patient education is associated with a rapid and sustained improvement in outcomes for people living with CM/MOH.

Some countries do not allow initiation of anti-CGRP treatment until MOH is addressed with different drug withdrawal approaches, which may be time- and resources-consuming [46–48]. Delaying effective treatment for MOH is highly detrimental to people living with this disabling disorder [7]. The present results of the RESOLUTION trial may suggest a benefit of starting eptinezumab simultaneously with patient education to obtain rapid and sustained improvements across multiple endpoints collected during the 24-week trial, and can potentially inform an optimized long-term preventive treatment strategy for managing CM and MOH in clinical practice. The RESOLUTION trial design may serve as a model for the design of future studies aimed towards rapid and sustained improvements in disease status in people living with high-frequency migraine complicated by medication overuse.

### Limitations

This two-arm trial was designed to assess the value of eptinezumab as an add-on to BEI. This trial did not include a full, 4-arm factorial design; specifically, the trial did not include an eptinezumab arm without BEI or an eptinezumab placebo arm without BEI. This design does not address the benefits of patient education alone, which has been previously demonstrated [43–45], and the benefits of eptinezumab without patient education in individuals with CM and MOH, which has also been demonstrated [20–22]. While the absence of an arm without BEI precludes definitive conclusions as to whether the sustained improvements seen during the OLE are driven by eptinezumab, carryover effects of patient education, or natural history of MOH resolution over time, we chose this design to adhere to the recommendation that individuals with CM and MOH should receive patient education [7, 8, 10, 13, 15, 16]. The present findings are also limited in their generalizability. Individuals with MOH who were not diagnosed with CM, including those with other primary or secondary headache disorders, were not included in this trial. The trial also excluded patients with barbiturate or opioid overuse or previous anti-CGRP treatment failures, as well as those with previous clinically significant psychiatric or cardiovascular diseases, limiting generalizability to such situations. In addition, 98% of trial participants were from a single geographic area (Europe). The relevance of the trial’s findings to other geographic areas that differ from Europe in acute headache medication use patterns and treatment strategies for MOH may be limited [7, 15, 16], including for North America where opioid overuse is prevalent among individuals with migraine [49, 50]. More studies are needed to determine how people living with CM and MOH in different settings with different medication availability and treatment traditions may benefit from preventive treatment with patient education. Lastly, as this trial did not include follow-up beyond 24 weeks, we are unable to determine whether the participants would benefit from more infusions or whether they would relapse into MOH if the treatment were interrupted.

### Conclusions

Results from the OLE of the RESOLUTION trial demonstrate that the efficacy seen with eptinezumab over the 12-week double-blind period was sustained for up to 24 weeks in adults with CM and MOH who also received patient education. During the placebo-controlled period, in comparison with the placebo plus BEI group, the eptinezumab plus BEI group experienced greater reductions in disease burden and improvements in patient-reported outcomes in the first 4 weeks and across Weeks 1–12. Improvements were sustained across Weeks 13–24 following a second eptinezumab infusion, with similar improvements in participants who switched from placebo to eptinezumab. Eptinezumab in combination with patient education was well-tolerated and no new safety signals were identified during the OLE. The rapid and sustained improvements observed over 24 weeks with eptinezumab treatment support two infusions of eptinezumab, combined with patient education, as a reasonable first approach for treating CM and MOH.

## Electronic supplementary material

Below is the link to the electronic supplementary material.

Supplementary material 1

## Data Availability

In accordance with EFPIA’s and PhRMA’s “Principles for Responsible Clinical Trial Data Sharing” guidelines, Lundbeck is committed to responsible sharing of clinical trial data in a manner that is consistent with safeguarding the privacy of patients, respecting the integrity of national regulatory systems, and protecting the intellectual property of the sponsor. The protection of intellectual property ensures continued research and innovation in the pharmaceutical industry. Deidentified data are available to those whose request has been reviewed and approved through an application submitted to https://www.lundbeck.com/global/our-science/clinical-data-sharing.

## Abbreviations

AE: adverse event
BEI: brief educational intervention
CGRP: anti–calcitonin gene-related peptide
CM: chronic migraine
eDiary: electronic diary
HIT-6: six-item Headache Impact Test
ICHD-3: International Classification of Headache Disorders 3rd edition
mAb: monoclonal antibody
MHDs: monthly headache days
MMDs: monthly migraine days
mMIDAS: modified Migraine Disability Assessment
MMRM: mixed model for repeated measures
MOH: medication-overuse headache
MSQ v2.1: Migraine-Specific Quality-of-Life questionnaire v2.1
OLE: open-label extension period
PGIC: Patient Global Impression of Change
PI-MBS: patient-identified most bothersome symptom
TEAE: treatment-emergent adverse event
WPAI:M: Migraine-specific Work Productivity and Activity Impairment questionnaire
VAS: visual analogue scale
