# Supplementary material for: Efficacy and safety of eptinezumab in adults with chronic migraine and medication-overuse headache who also received patient education: 24-week results of the randomized RESOLUTION trial
Source: J Headache Pain. 2026 Jun 13;27(1):157. doi: 10.1186/s10194-026-02423-x (PMC13274074; doi:10.1186/s10194-026-02423-x)
Supplement: Supplementary file 1 — Supplementary material 1 [file 10194_2026_2423_MOESM1_ESM.pdf]

Supplementary Material

Table 1. Demographics and baseline characteristics of participants treated in the open-label extension period (all-participants-treated-open-label set).

		**Eptinezumab/ Eptinezumab[a] (N=300)**	**Placebo/ Eptinezumab[a] (N=293)**
**Demographics[b]**	Age (years), mean (SD)	45.7 (11.97)	45.0 (12.04)
	Sex, n (%)		
	Female	261 (87.0%)	247 (84.3%)
	Male	39 (13.0%)	46 (15.7%)
	Geographic region, n (%)		
	Europe	293 (97.7%)	287 (98.0%)
	Australia	5 (1.7%)	4 (1.4%)
	United States	2 (0.7%)	2 (0.7%)
**Baseline clinical characteristics,[c] mean (SD)**	MMDs	21.0 (4.26)	20.9 (4.30)
	MHDs	21.8 (3.92)	21.7 (3.97)
	Monthly days with acute medication use[d]	20.1 (4.26)	20.1 (4.54)
	Monthly days with triptan use[d]	13.3 (7.63)	14.0 (7.72)
	Monthly days with non-opioid analgesic or NSAID use[d]	11.4 (8.74)	11.6 (8.79)
**Baseline PRO scores,[e] mean (SD) [N]**	HIT-6 total score	66.4 (5.01) [288]	66.5 (5.05) [279]
	mMIDAS total score[f]	33.3 (19.64) [286]	29.7 (19.76) [277]
	WPAI:M domain score: Absenteeism	19.1 (28.74) [193]	14.1 (23.72) [191]
	WPAI:M domain score: Presenteeism	56.5 (21.15) [181]	57.8 (21.00) [185]
	WPAI:M domain score: Work productivity loss	60.5 (22.82) [181]	61.2 (22.00) [185]
	WPAI:M domain score: Activity impairment	62.2 (20.07) [285]	62.6 (20.23) [270]
	MSQ v2.1 domain score: Role function-restrictive	34.3 (17.51) [285]	35.0 (19.02) [271]
	MSQ v2.1 domain score: Role function-preventive	52.1 (22.07) [285]	51.4 (22.42) [271]
	MSQ v2.1 domain score: Emotional function	42.9 (25.79) [285]	41.3 (26.49) [271]
	EQ-5D-5L VAS score	65.4 (20.12) [285]	67.3 (19.26) [270]

[a] All participants received brief educational intervention before eptinezumab or placebo infusion at baseline; at the end of Week 12, all participants received eptinezumab.

[b] Data were collected at the first screening visit.

[c] Baseline values were calculated from the eDiary during the screening period.

[d] Participants may have taken more than one type of medication. Opioid analgesics were only permitted up to 4 days per month. A total of four participants used ergotamine during the screening period.

[e] Baseline values were calculated from electronic PRO measures completed prior to infusion at the baseline visit.

[f] The mMIDAS has a one-month recall period and is a modified version of the original MIDAS, which has a three-month recall period.

eDiary, electronic diary; HIT-6, 6-item Headache Impact Test; MHDs, monthly headache days; MMDs, monthly migraine days; MIDAS, Migraine Disability Assessment; mMIDAS, modified Migraine Disability Assessment; MSQ v2.1, Migraine-Specific Quality of Life questionnaire (version 2.1); NSAID, nonsteroidal anti-inflammatory drug; PRO, patient-reported outcome; SD, standard deviation; VAS, visual analogue scale; WPAI:M, Migraine-specific Work Productivity and Activity Impairment questionnaire.

Supplemental Table 2. Summary of scores for electronic patient-reported outcomes at Week 24 (all-participants-treated-open-label set).

	**Eptinezumab/ Eptinezumab[a]**	**Placebo/ Eptinezumab[a]**
**PGIC score**		
LS mean (SE) [N]	2.4 (0.10) [283]	2.4 (0.10) [277]
**PI-MBS score**		
LS mean (SE) [N]	2.7 (0.10) [283]	2.7 (0.10) [277]
**HIT-6 total score**		
Change from baseline, LS mean (SE) [N]	-7.5 (0.60) [274]	-7.1 (0.60) [265]
**mMIDAS total score**		
Change from baseline, LS mean (SE) [N]	-15.8 (1.33) [270]	-16.3 (1.33) [263]
**WPAI:M absenteeism sub-score**		
Change from baseline, LS mean (SE) [N]	-6.9 (1.96) [161]	-4.8 (1.98) [145]
**WPAI:M presenteeism sub-score**		
Change from baseline, LS mean (SE) [N]	-18.3 (2.57) [152]	-20.3 (2.61) [136]
**WPAI:M work productivity loss sub-score**		
Change from baseline, LS mean (SE) [N]	-19.1 (2.70) [152]	-21.3 (2.75) [136]
**WPAI:M activity impairment sub-score**		
Change from baseline, LS mean (SE) [N]	-18.2 (2.08) [268]	-19.8 (2.09) [256]
**MSQ v2.1 role function-restrictive domain score**		
Change from baseline, LS mean (SE) [N]	24.9 (1.84) [270]	25.5 (1.84) [257]
**MSQ v2.1 role function-preventive domain score**		
Change from baseline, LS mean (SE) [N]	20.0 (1.76) [270]	20.0 (1.76) [257]
**MSQ v2.1 emotional function domain score**		
Change from baseline, LS mean (SE) [N]	24.4 (2.05) [270]	24.0 (2.05) [257]
**EQ-5D VAS score**		
Change from baseline, LS mean (SE) [N]	5.0 (1.56) [268]	4.1 (1.57) [256]

[a]All participants received brief educational intervention before eptinezumab or placebo infusion at baseline; at the end of Week 12, all participants received eptinezumab. Mixed models for repeated measures were used.

The mMIDAS has a one-month recall period and is a modified version of the original MIDAS, which has a three-month recall period.

HIT-6, 6-item Headache Impact Test; LS, least-squares; MIDAS, Migraine Disability Assessment; mMIDAS, modified Migraine Disability Assessment; MSQ v2.1, Migraine-Specific Quality of Life (version 2.1); PGIC, Patient Global Impression of Change; PI-MBS, patient-identified most bothersome symptom; SE, standard error; VAS, visual analogue scale; WPAI:M, Migraine-specific Work Productivity and Activity Impairment questionnaire.

Supplemental Figure 1. Trial design.

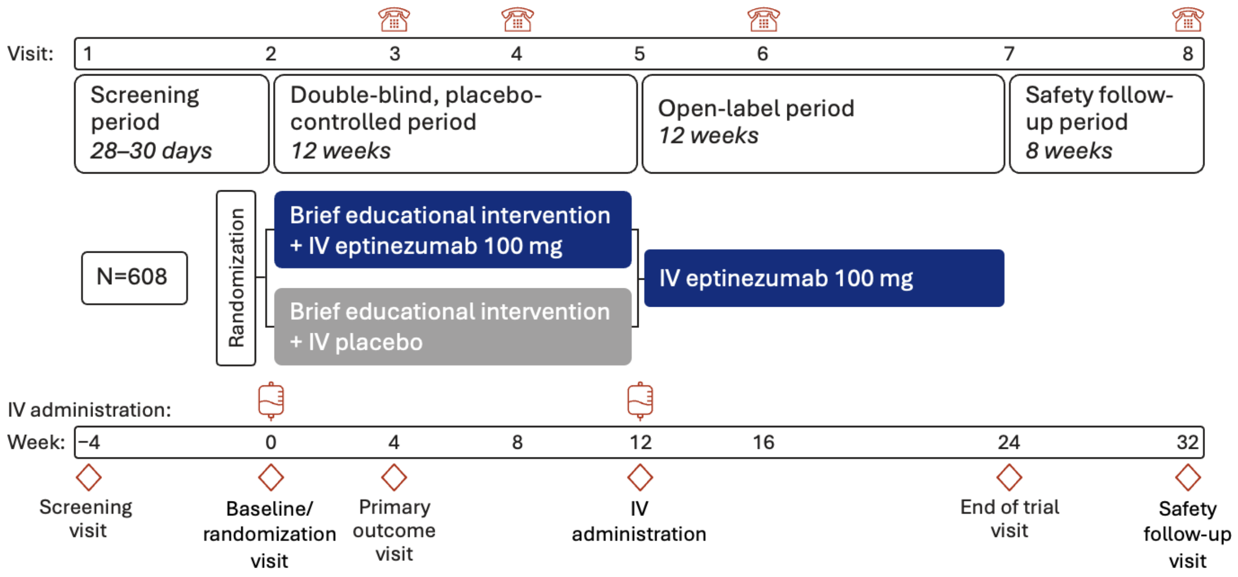

After the screening period, eligible participants were randomized 1:1 to receive IV infusion of eptinezumab 100 mg or placebo at the baseline visit (Day 0), with all receiving an ~10-minute brief educational intervention prior to infusion about medication overuse and how to stop or reduce the overuse of acute medications. At the end of Week 12, all participants received eptinezumab 100 mg during the open-label extension period.

IV, intravenous.

Supplemental Figure 2. Change from baseline in MHDs over **(A)** 4-week and **(B)** 12-week intervals (all-participants-treated-open-label set).

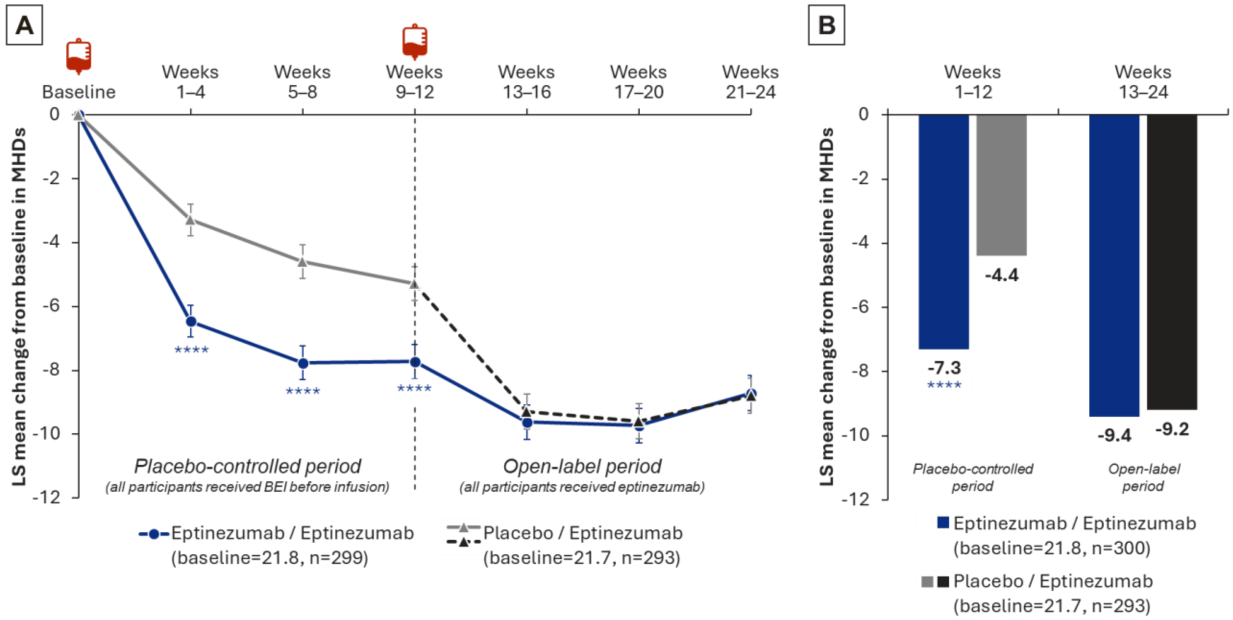

All participants received brief educational intervention before eptinezumab or placebo infusion at baseline; at the end of Week 12, all participants received eptinezumab. Error bars show standard error, and a mixed model for repeated measures was used. *Post hoc p*-values for differences between eptinezumab and placebo are presented for the placebo-controlled period. ****p<0.0001 vs placebo.

BEI, brief educational intervention; LS, least-squares; MHDs, monthly headache days.

Supplemental Figure 3. Participants no longer meeting the thresholds for **(A)** CM or MOH, **(B)** CM, or **(C)** MOH criteria over 4-week intervals (all-participants-treated-open-label set).

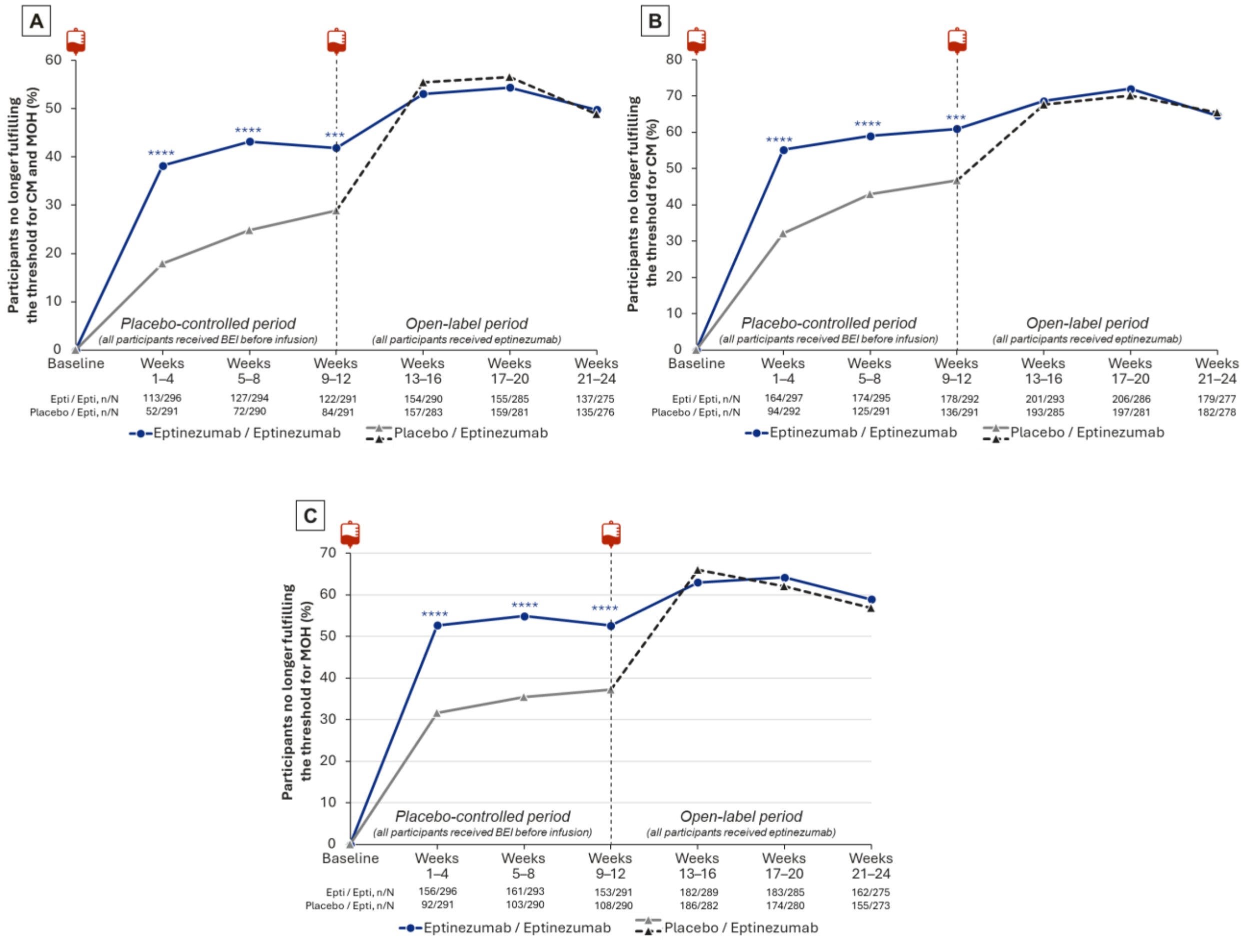

All participants received brief educational intervention before eptinezumab or placebo infusion at baseline; at the end of Week 12, all participants received eptinezumab. Thresholds defining CM and MOH were based on ICHD-3 diagnostic criteria for each disease, with CM determined by the monthly frequency of headache days (≥15) and migraine days (≥8) and MOH determined by the monthly frequency of acute medication use (≥10 or ≥15 days depending on medication class). *Post hoc p*-values for differences between eptinezumab and placebo are presented for the placebo-controlled period. ***$p<0.001$, ****$p<0.0001$; both vs placebo.

BEI, brief educational intervention; CM, chronic migraine; Epti, eptinezumab; ICHD-3, International Classification of Headache Disorders, 3rd edition; MOH, medication-overuse headache.

Supplemental Figure 4. Change from baseline in WPAI:M sub-scores (all-participants-treated-open-label set).

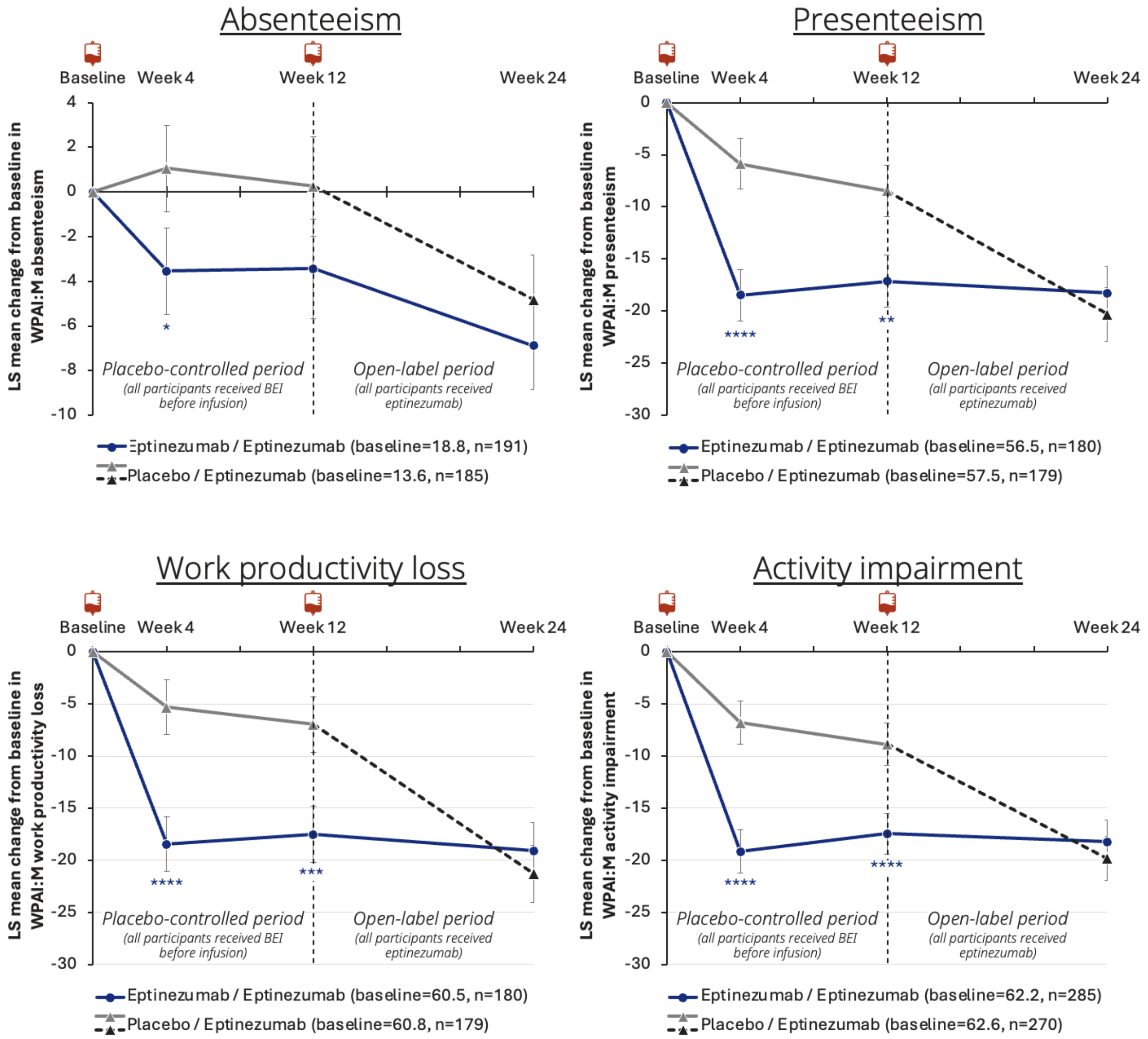

All participants received brief educational intervention before eptinezumab or placebo infusion at baseline; at the end of Week 12, all participants received eptinezumab. Error bars show standard error, and mixed model for repeated measures was used. *Post hoc p*-values for differences between eptinezumab and placebo are presented for the placebo-controlled period; where a *p*-value is not presented for the placebo-controlled period, the difference was not significant. *$p<0.05$, **$p<0.01$, ***$p<0.001$, ****$p<0.0001$; all vs placebo.

BEI, brief educational intervention; LS, least-squares; WPAI:M: Migraine-specific Work Productivity and Activity Impairment questionnaire.
